# The Microbiome in Health and Disease from the Perspective of Modern Medicine and Ayurveda

**DOI:** 10.3390/medicina56090462

**Published:** 2020-09-11

**Authors:** Robert Keith Wallace

**Affiliations:** Department of Physiology and Health, Maharishi International University, Fairfield, IA 52556, USA; kwallace@miu.edu

**Keywords:** microbiome, Ayurveda, gut bacteria, diet, lifestyle, disease, prevention, integrative medicine

## Abstract

The role of the microbiome in health and disease helps to provide a scientific understanding of key concepts in Ayurveda. We now recognize that virtually every aspect of our physiology and health is influenced by the collection of microorganisms that live in various parts of our body, especially the gut microbiome. There are many external factors which influence the composition of the gut microbiome but one of the most important is diet and digestion. Ayurveda and other systems of traditional health have for thousands of years focused on diet and digestion. Recent research has helped us understand the connection between the microbiome and the many different prevention and therapeutic treatment approaches of Ayurveda.

## 1. Introduction

DNA sequencing technology and bioinformatics have made it possible to evaluate the composition of the diverse community of bacteria, archaea, fungi, viruses, and other organisms that form the microbiome. A growing body of research has now correlated the microbiome with a wide variety of diseases [1,2,3,4]. The microbiome is present in many parts of the body but the largest collection of over 30 trillion bacteria is in the gut [5].

The gut microbiome participates in vital processes including digestion, energy homeostasis and metabolism, the synthesis of vitamins and other nutrients, and the development and regulation of immune function. It also contributes to the production of numerous compounds that enter the blood and affect various tissues and organs of the body [3,4].

An important consideration is the recognition of the enormous variation in the gut microbiota composition in each individual, as well as in each area of the digestive tract. Throughout the intestines there are specific niches that house individual microbial communities, which can be immunologically more active than others. A balanced and diverse microbiome is critical for maintaining health and immunological balance [6,7].

When the microbiome is in balance it contributes to many health benefits, but when out of balance, it can cause problems in the gut and other areas of the body. Dysbiosis arises when the delicate and elaborate ecology of microbial communities are disrupted by internal or external factors. A disrupted microbiome is characterized by the overgrowth of one or more of the different microbial colonies. A complex interaction between the microbiome and immune systems may result in an inflammatory state [8,9]. An imbalanced microbiome has been associated with a number of gastrointestinal diseases including irritable bowel syndrome (IBS) and inflammatory bowel disorder (IBD) [10,11,12]. Conditions such as asthma, atopy, childhood obesity, and autism spectrum have been correlated with excess antibiotic use and a resulting alteration in the microbiome in childhood [13,14,15,16]. Numerous other conditions such as obesity, autoimmune disorders, cardiovascular disease, cancer, and neurological disorders have also been linked with changes in the microbiome [1,2,3,4,17,18,19].

Diet can rapidly alter the composition of the microbiome [20,21,22,23]. A western diet high in meat products, providing nutrients such as choline and carnitine, can cause certain gut bacteria to produce trimethylamine (TMA). TMA is absorbed into the bloodstream and then oxidized in the liver to form trimethylamine-N-oxide (TMAO). High levels of TMAO have been suggested to contribute to cardiovascular disease by interfering with cholesterol metabolism and transportation, foam cell formation, and platelet aggregation [24,25,26,27,28,29,30,31,32,33,34].

Gut bacteria may also affect cardiovascular disease by a decrease in fiber intake. Dietary fiber is a rich source of food for gut bacteria and its reduction can lead to a decreased bacterial production of the short chain fatty acid butyrate. This change can lead to dysbiosis and local inflammation in the gut lining, resulting in impaired gut barrier function and the possible leakage of bacterial toxins, such as lipopolysaccharides, into the bloodstream [30,31,32,33,34].

There are other microorganism communities throughout the body which can contribute to health and disease. At one time, the lung was considered a sterile organ, but we now recognized that it has its own microbiome which extends into the lower lung. There is cross talk between the lung and gut microbiomes, which could be relevant to patients with COVID 19 that display gastrointestinal symptoms [35,36,37,38].

The gut microbiome also affects the brain and mental health. The basis for this interaction is the gut–brain axis, which consists of the brain, immune system, endocrine system, enteric nervous system (ENS), enteroendocrine system (EEC), and the gut bacteria. There is a bidirectional flow of information between the gut and brain. The most direct is through the vagus nerve, which is an important and long nerve in the body that regulates many internal functions. A less direct means of communication is through different chemical messengers, such as neurotransmitters, hormones, and peptides. The gut produces numerous peptides and neurotransmitters. Many of these are also found in the brain. The secretion of these gut-derived chemicals can be influenced by the composition of the gut microbiome. In addition, the gut microbiome can also produce its own unique array of chemical messengers, that go into the bloodstream and affect different parts of the body. There is also research showing that gut microbes can activate immune cells in the gut wall, which causes the release of proinflammatory cytokines and ultimately may affect the permeability of the blood–brain barrier [39,40,41]. Animal studies have shown that a disrupted microbiome can cause anxiety-like and depression-like behaviors [42,43]. A new field of psychobiotics has even emerged, which utilizes probiotics to affect moods and behavior in humans [44].

While the precise manner in which the microbiome participates in these many disease states is still not completely clear, there are currently a number of therapeutic approaches that are now being tested in clinical trials including diet, prebiotics, probiotics, antibiotics, and fecal microbiome transplantation (FMT) [45,46,47]. Recent studies, for example, have utilized personalized nutritional advice based on microbiome data and other factors [48,49,50]

## 2. Ayurveda

Hippocrates, the father of modern western medicine, is famous for his expression “All disease begins in the gut.” Ayurveda places great importance on proper diet and digestion, as well as on all aspects of lifestyle. Some researchers consider Ayurveda to be an ancient science of epigenetics [51]. The Ayurvedic practitioners might not have understood the precise nutrigenomic mechanics of how food can affects gene expression [52], but they did recognize that each individual has his or her own unique psychophysiological constitution, which is affected by diet, digestion, lifestyle, stress management, and environmental factors [53].

Ayurveda describes the functioning of our mind and body in terms of three main doshas—the governing principles of the physiology—Vata, Pitta, and Kapha [54]. In a previous paper, we have reviewed the research on the physiological, biochemical, and genetic correlates of each of the doshas. As we explain, the category of Vata dosha includes processes responsible for movement at all levels of the physiology, excretion of wastes, and also cognition. The primary location of Vata, according to Ayurveda, is in the colon, where most of the gut microbiome is located. Pitta dosha is responsible for such functions as digestion, metabolism, thermoregulation, and energy homeostasis and resides in the mid gut area. Kapha dosha governs the growth and maintenance of structure and its primary location is in the chest area. Genetic research shows a significant difference between each dosha type and gene expression. Genes related to cell cycles were upregulated in the Vata types whereas genes in the immune response pathways were upregulated in the Pitta type, and genes in the immune signaling pathways were upregulated in Kapha types. Each dosha type is suggested to display its own style of brain functioning [55,56]. In Ayurveda, one of the first stages of an assessment of a person’s health is to determine the distribution of the three doshas at birth, which is called Prakriti. In addition, an assessment is made of the state of balance of the doshas, which is called Vikriti. In fact, one can have a dominant Vata Prakrti at birth, but at the time of the diagnosis, the primary imbalance may be in Pitta due to some environmental factor.

**Figure 1:**
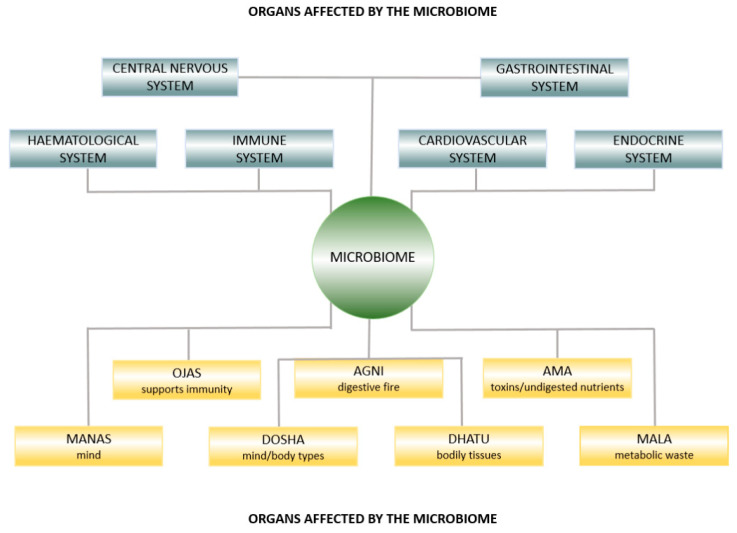
Ayurvedic anatomy affected by the microbiome.

In Figure 1, we illustrate that the microbiome is integral to both modern medicine and Ayurveda. We elaborate on this concept throughout the article.

## 3. Prakriti and Gut Bacteria

One study has examined the relationship between Prakriti and the composition of the microbiome. The researchers found that three main Prakriti types, Vata, Pitta, or Kapha, had a unique microbiome composition [57]. The population studied were from the same region and had similar dietary habits. The main bacteria of all the subjects were from the phyla Bacteroidetes and Firmicutes. There were distinct differences between the Vata, Pitta, or Kapha groups in less common bacteria. The extreme Pitta individuals, for example, had more butyrate producing microbes which might help protect them from inflammatory diseases. The extreme Kapha women had larger amounts of a type of bacteria called *Prevotella copri*, which has been associated with patients who have rheumatoid arthritis and insulin resistance. A more recent paper analyzes how the concept of Prakriti can be used as a stratifier of the gut microbiome [58].

## 4. Ayurveda Herbs and Spices and the Microbiome

Ayurveda considers food as medicine. It uses spices and herbs to help create and maintain balance in the physiology, and to treat specific disorders. Recent research shows that both ginger and a herbal preparation called Triphala can have beneficial effects on the microbiome. Triphala consists of three fruits: Emblica officinalis (Amalaki), Terminalia bellerica (Bibhitaki), and Terminalia chebula (Haritaki). It is a fundamental component of Ayurvedic gastrointestinal and rejuvenation treatment programs, particularly to help improve elimination. Studies have found that Triphala has different potential clinical applications which include appetite stimulation and a reduction in hyperacidity and constipation. It also has a number of inherent biological properties such as anti-inflammatory, immunomodulatory, antibacterial, antimutagenic, adaptogenic, hypoglycemic, antineoplastic, chemoprotective and radioprotective, and antioxidant activities. In the study, it was shown that the polyphenols in Triphala modulate the human gut microbiome and thereby promote the growth of beneficial Bifidobacteria and Lactobacillus while inhibiting the growth of undesirable gut microbes. The authors also suggest that the bioactivity of Triphala is elicited by gut microbiota that generate a variety of anti-inflammatory compounds [59].

Turmeric is another Ayurvedic preparation which has been found to affect digestion and the microbiome. Turmeric and its active ingredient curcumin have been the subject of thousands of peer-reviewed and published biomedical studies, with hundreds of potential preventive and therapeutic applications on such diseases as ulcerative colitis, stomach ulcers, osteoarthritis, heart disease, cancer, and neurodegenerative disorders [60,61]. Interestingly, the active ingredient of turmeric, curcumin, has been shown to be biological active but because of its poor bioavailability and inability to reach target tissues, it has not been found to be effective in clinical trials [62,63]. It may also be ineffective because we no longer have the synergic effects of the other ingredients. It is suggested that curcumin may restore dysbiosis of the gut microbiome and as a result have a neuroprotective effect, however, future research is needed to know how curcumin actually affects the microbiome in different individuals [64,65].

There are many other important Ayurveda herbs and preparations that need to be further studied. One of the most important categories are Rasayanas which are designed to lengthen the lifespan [66]. Once again, future research will be needed to examine the specific effects of Rasayana on the microbiome.

## 5. Ama and Leaky Gut Syndrome

Ayurveda explains that most diseases are caused by an accumulation of ama or undigested food. Ama literally means “uncooked food,” but it can be understood from a scientific perspective as endogenous toxins resulting from imbalanced or incomplete digestion. Ama can be formed as a result of reduced Agni, or digestive power. Agni has a number of different meanings and not only relates to digestive enzymes but also to the metabolic process in the different tissues or dhatus of the body [67]. Ama is initially formed in the digestive tract, but at a later stage of disease it can leak into the bodily tissues and turn into Amavisha, a reactive form of ama, that leads to tissue disruption and chronic inflammation and disease.

It has been suggested that compromised mucosal integrity, such as a disruption in the tight junctions, leads to dysbiosis, resulting in the formation of Ama [68]. Ama is also produced at other levels of the physiology, including the cellular level. Excessive formation of free radicals contributes to the formation of Ama. A variety of free radicals and reactive oxygen species (ROS) are produced during cellular metabolism. Excessive amounts of these reactive molecules can cause damage, starting the disease process. The ability to control their concentrations may be helpful for the prevention and treatment of many disorders.

Antioxidants “scavenge” free radicals and ROS and render them harmless. Antioxidants can be lipid- or water-soluble; some are produced in the body and others are obtained from food or dietary supplements. Natural antioxidants range from vitamins to enzymes to herbal mixtures. Powerful antioxidants are present in the bioflavonoids found in concentrated form in Rasayanas. The use of these Rasayanas, might be helpful in neutralizing the excessive free radical activity that contributes to Ama formation. However, it is not clear if accumulated Ama in the body can be removed by the use of Rasayanas and antioxidants alone. Other Ayurvedic methodologies may be required to eradicate accumulated Ama. Rasayanas are best administered after a detoxification treatment such as panchakarma and are utilized to neutralize ongoing damage to the physiology and regenerate the system [68].

It is interesting to interpret the meaning of Ama in terms of recent findings. Remarkable progress has been made in understanding the mechanisms of leaky gut syndrome in celiac patients. In celiac disease, the tight junctions that hold the cells of the gut wall together become loose, and as a result, undigested food and harmful substances “leak” through the gut wall and into the bloodstream, causing inflammation. The exact mechanism of this process involves a product of gluten called gliadin, which interacts with receptors on the surface of the cells in the small intestine, causing the production of zonulin. Zonulin then causes the proteins, which bind tight junctions, to relax and allow unwanted substances to enter the bloodstream. In celiac patients, this process is exaggerated and can ultimately result in a harmful autoimmune reaction [69,70]. A disruption in the gut microbiome has been associated with problems in the gut barrier. It is suggested that gut dysbiosis may cause the release of zonulin but the exact mechanisms are complex and still under investigation [71,72].

## 6. Biorhythms and Gut Bacteria

Ayurveda clearly identifies daily, seasonal, and lifetime rhythms. Each day, for example, consists of a sequence of periods, which are characterized by Vata, Pitta, or Kapha. The day starts with a Kapha period from 6 am to 10 am, then a Pitta period from 10 am to 2 pm, and then a Vata period from 2 pm to 6 pm. Next is another Kapha period from 6 pm to 10 pm, a Pitta from 10 pm to 2 am and finally a Vata from 2 am to 6 am. Each season is represented by either one dosha or a combination of doshas: late autumn and winter are cold and dry and correspond to the Vata dosha. Summer is hot and naturally corresponds to Pitta dosha. Spring is cold and wet and corresponds to Kapha dosha. The daily routine is called dinacharya and the seasonal routine is called ritucharya.

Modern medicine also recognizes the importance of daily rhythms to your health and the 2017 Nobel Prize in Physiology and Medicine was awarded for research on the genetic basis of biological rhythms. From bacteria to humans, almost all forms of life have an internal “biological clock,” which maintains an approximately 24 h rhythm. When external signals of light and dark are introduced unnaturally, the master clock becomes confused and this creates health problems. Shift workers, for instance, have been shown to have a higher incidence of cancer, cardiovascular disease, digestive disorders, and obesity, as well as psychiatric and neurodegenerative diseases [73].

One of the most important timing issues for the body, which has been seen in research on animals and humans, is when you eat. If you eat within 2 h before you normally go to sleep, it can desynchronize the circadian rhythms of certain cells in the intestine and liver from those in the rest of your body [74].

Gut bacteria also have biological rhythms. One study created jet lag in mice by forcing these normally nocturnal animals to stay awake during the day. When the researchers transferred the gut bacteria from jet-lagged mice into germ-free mice, the recipient mice developed both obesity and glucose intolerance [75]. A number of studies have shown that time-restricted feeding and eating frequency affects the composition of the microbiome [76]. Recent research on the gut bacteria reveals not only the presence of a daily rhythm, but also seasonal biorhythms [77,78]. The relationship between the circadian rhythms and gut microbiota appears to be bidirectional and may have important influences on health [79,80,81,82].

Ayurvedic recommendations of specific diets at specific times may relate to the seasonal rhythms of the microbiome. Over the winter, for example, Kapha and Ama are said to build up in the body. Early spring is described as the ideal time to rebalance Kapha and reduce Ama to prevent toxins and excess mucus from creating congestion and allergies. Foods that are primarily Kapha in nature—heavy, greasy, and mucus forming—tend to increase both Kapha and Ama. Ayurveda recommends reducing or eliminating Kapha foods during this time, which might help to heal the gut and reboot the microbiome [83].

## 7. Probiotic Enemas and Bastis

Probiotic enemas have been suggested to help cure certain neurological disorders [84]. Probiotics that are taken orally must pass through your stomach and the small intestine, which is not ideal because stomach acid and digestive enzymes destroy many of the valuable probiotic bacteria long before they reach the colon. A probiotic enema, however, provides an almost instantaneous route to the colon, where most of the gut bacteria live.

The Ayurvedic term for enema is basti, and bastis are a valuable part of the deep purification and detox treatment program known as panchakarma, which, as we mentioned, cleanses the body of impurities and promotes health and longevity. There are many types of bastis: some are for purification, others for elimination, others strengthen the tissues and provide valuable nutrients. Bastis use ingredients such as sesame oil, medicated ghee, buttermilk, lassi, many different combinations of herbs, and in special cases, bone broth. A search of Pub Med shows a number of published studies on different types of bastis that have been used for various clinical applications [85,86,87,88,89,90,91,92].

These studies, however, do not mention the use of bastis as a means to modify or reboot the gut bacteria. By studying how bastis affect gut bacteria composition we might gain a better scientific understanding of the mechanisms of their beneficial actions on specific health conditions. We already know that sesame oil has a positive effect on colon cells, and on certain beneficial bacteria [93,94].

In a more recent study, the metabolomic profile was taken after a 6-day panchakarma treatment. The experimental group consisted of healthy male and female subjects whose treatment program included herbs, vegetarian diet, meditation, yoga, and massage. The results showed that 12 plasma phosphatidylcholines decreased after treatment in the experimental group as compared to the control group of 54 subjects. There were changes in metabolites across many pathways such as phospholipid biosynthesis, choline metabolism, and lipoprotein metabolism, with statistically significant changes in the plasma levels of phosphatidylcholines, sphingomyelins and others in just 6 days. It is unclear whether the lipid metabolites were modulated by the gut microbiome or by the external agents such as herbs, vegetarian diet, meditation, yoga, and massage [95].

The study of panchakarma and other Ayurvedic purification treatments is an important area of future research. It would also be useful to compare the modern use of fecal transplant treatment with all the different types of basti treatments.

## 8. The Gut–Brain Axis and Ojas

The gut–brain axis, as we described earlier, consists of a number of major systems in the body, as well as the gut bacteria. The brain communicates to the gut through either nerves or through hormones and chemical messengers. The gut–brain axis includes the enteric nervous system (ENS), enteroendocrine system (EEC) and the gut bacteria, which all produce different chemicals. The ENS produces more than 30 neurotransmitters. Almost 90% of all the serotonin in the body is produced by cells in the gut, as well as 50% of the dopamine [39,40,41,96].

According to Ayurveda, the digestion and gut play leading roles in immunity. Ayurveda speaks about a substance or a process called Ojas, the finest product of healthy digestion, which strengthens the immune system and has many beneficial effects on the mind and body.

Can Ojas be identified by modern science? In Ayurveda there is a description of seven basic tissues or dhatus and three waste products or malas. The tissues roughly correspond to the tissues as discussed in modern physiology and the waste products include feces, urine, and sweat. The tissues are important because digestion includes the transformation of food into each of these tissues and ultimately to Ojas. It is unclear what this means in terms of modern science. It might correspond to a metabolic process that involves specific precursors which come from a well-functioning digestive system and a balance microbiome.

There are several possible candidates for Ojas. One is serotonin, a key regulator of mood, sleep, appetite, and other brain functions. As we mentioned, the gut produces most of the serotonin in the body, which circulates throughout your bloodstream and influences not only your immune system, but your heart rate, blood clotting, intestinal motility, pulmonary arteries, heart, brain, and mammary glands, as well as the cell growth of liver and bone cells [97]. One of the key precursors of serotonin is tryptophan, an essential amino acid, which is obtained through the normal digestion of certain foods.

Ojas is described as being beneficial to all parts of the body so we might ask if excess serotonin has any deleterious effects. An overabundance of serotonin due to drugs can lead to a number of damaging symptoms, including anxiety, sweating, sleep disturbance, appetite changes, headaches, confusion and lethargy, dilated pupils, rapid heart rate, etc. [98]. Additionally, individuals with social phobia have been shown to have excess serotonin [99].

Another interesting candidate is a chemical called butyric acid, which, as we mentioned, is a by-product of the gut bacteria and has numerous beneficial effects, including improvement of immunity [100].

Ojas is described in the Ayurvedic texts as having many properties, such as its ability to coordinate the junction between the mind and body. Some of these effects may not be covered by either serotonin or butyric acid. The exact nature of Ojas remains an ongoing research project.

## 9. Stress, Ayurveda, and Psychobiotics

Stress can have many deleterious effects. When the brain triggers the adrenal glands to release cortisol, it goes into the bloodstream and affects both the gut and the gut bacteria. Cortisol can increase the intestinal permeability, which results in a leaky gut. It can also shut down the activity of the gut immune system [71,101].

Ayurveda uses a wide variety of approaches to improve the mind or manas and to alleviate the effects of stress. One of these is meditation, which is an integral part of both Ayurveda and its sister discipline, yoga. The Transcendental Meditation (TM) program introduced by Maharishi Mahesh Yogi over fifty years ago has been shown to have many distinct physiological changes, including a reduction in cortisol, as well as beneficial effects on mental and physical health [102,103,104,105,106,107]. To date, we know of no studies which have examined the effect of meditation on the gut microbiome.

Ayurveda has long explained that disturbances in our mental state, which are usually associated with a Vata imbalance, can be traced to problems in the nervous system and the gut. It recommends specific herbs such as Ashwagandha and Brahmi, which may interact with the microbiome. It also has a long tradition of using natural probiotics such as lassi. Modern research on the microbiome validates this concept, showing that depression and anxiety may be linked to a disrupted microbiome and may be improved through the use of probiotics.

One brain imaging study showed that women react differently to stimuli depending on the type of bacteria they have in their gut. The same researchers had previously shown that diet could affect the brain. They gave subjects either a psychobiotic (a probiotic mixture that is used for mental health) or a placebo. They then showed the subjects images of frightened faces while measuring brain activity. The subjects taking the placebo showed a normal stress response with specific areas of the brain responsible for emotions being activated. Subjects receiving the psychobiotic showed a reduced stress response in these same areas of the brain [108,109].

## 10. The Future of Ayurveda and Modern Medicine

We are living in a transitional moment in medicine. New studies are emerging that will help us make better choices. The field of integrative medicine represents an important achievement since it combines the best of modern medicine with the best of traditional system of natural health, such as Ayurveda. Integrative medicine benefits greatly from research on the microbiome because it helps us better understand the ancient practices of Ayurveda in the light of modern science [110].

## References

[1] Zheng D., Liwinski T., Elinav E. (2020). Interaction between microbiota and immunity in health and disease. Cell Res..

[2] Malla M.A., Dubey A., Kumar A., Yadav S., Hashem A., Abd Allah E.F. (2019). Exploring the Human Microbiome: The Potential Future Role of Next-Generation Sequencing in Disease Diagnosis and Treatment. Front. Immunol..

[3] Mohajeri M.H., Brummer R.J.M., Rastall R.A., Weersma R.K., Harmsen H.J.M., Faas M., Eggersdorfer M. (2018). The Role of the Microbiome for Human Health: From Basic Science to Clinical Applications. Eur. J. Nutr..

[4] Armour C.R., Nayfach S., Pollard K.S., Sharpton T.J. (2019). A Metagenomic Meta-Analysis Reveals Functional Signatures of Health and Disease in the Human Gut Microbiome. mSystems.

[5] Sender R., Fuchs S., Milo R. (2016). Revised Estimates for the Number of Human and Bacteria Cells in the Body. PLoS Biol..

[6] Tong M., Li X., Parfrey L.W., Roth B., Ippoliti A., Wei B., Borneman J., Mcgovern D.P.B., Frank D.N., Li E. (2013). A Modular Organization of the Human Intestinal Mucosal Microbiota and Its Association with Inflammatory Bowel Disease. PLoS ONE.

[7] Huttenhower C., Gevers D., Knight R., Abubucker S., Badger J.H., Chinwalla A.T., Creasy H.H., Earl A.M., FitzGerald M.G., Fulton R.S. (2012). Structure, Function and Diversity of the Healthy Human Microbiome. Nature.

[8] Sommer F., Backhed F. (2015). The gut microbiota engages different signaling pathways to induce Duox2 expression in the ileum and colon epithelium. Mucosal Immunol..

[9] Geuking M.B., Köller Y., Rupp S., McCoy K.D. (2014). The interplay between the gut microbiota and the immune system. Gut Microbes.

[10] De Palma G., Collins S.M., Bercik P. (2014). The microbiota-gut-brain axis in functional gastrointestinal disorders. Gut Microbes.

[11] Kennedy P.J., Cryan J.F., Dinan T.G., Clarke G. (2014). Irritable bowel syndrome: A microbiome-gut-brain axis disorder?. World J. Gastroenterol..

[12] Khan I., Ullah N., Zha L., Bai Y., Khan A., Zhao T., Che T., Zhang C. (2019). Alteration of Gut Microbiota in Inflammatory Bowel Disease (IBD): Cause or Consequence? IBD Treatment Targeting the Gut Microbiome. Pathogens.

[13] Francino M.P. (2016). Antibiotics and the Human Gut Microbiome: Dysbioses and Accumulation of Resistances. Front. Microbiol..

[14] Mueller N.T., Whyatt R., Hoepner L., Oberfield S., Dominguez-Bello M.G., Widen E.M., Hassoun A., Perera F., Rundle A. (2015). Prenatal exposure to antibiotics, cesarean section and risk of childhood obesity. Int. J. Obes..

[15] Arrieta M.C., Stiemsma L.T., Amenyogbe N., Brown E.M., Finlay B. (2014). The intestinal microbiome in early life: Health and disease. Front. Immunol..

[16] Kozyrskyj A.L., Bahreinian S., Azad M.B. (2011). Early life exposures: Impact on asthma and allergic disease. Curr. Opin. Allergy Clin. Immunol..

[17] Burcelin R. (2016). Gut microbiota and immune crosstalk in metabolic disease. Mol. Metab..

[18] Patterson E., Ryan P.M., Cryan J.F., Dinan T.G., Ross R.P., Fitzgerald G.F., Stanton C. (2016). Gut microbiota, obesity and diabetes. Postgrad. Med. J..

[19] Sha S., Ni L., Stefil M., Dixon M., Mouraviev V. (2020). The human gastrointestinal microbiota and prostate cancer development and treatment. Investig. Clin. Urol..

[20] Conlon M.A., Bird A.R. (2015). The Impact of Diet and Lifestyle on Gut Microbiota and Human Health. Nutrients.

[21] Sakkas H., Bozidis P., Touzios C., Kolios D., Athanasiou G., Athanasopoulou E., Gerou I., Gartzonika C. (2020). Nutritional Status and the Influence of the Vegan Diet on the Gut Microbiota and Human Health. Medicina.

[22] Redondo-Useros N., Nova E., González-Zancada N., Díaz L.E., Gómez-Martínez S., Marcos A. (2020). Microbiota and Lifestyle: A Special Focus on Diet. Nutrients.

[23] Matsushita M., Fujita K., Nonomura N. (2020). Influence of Diet and Nutrition on Prostate Cancer. Int. J. Mol. Sci..

[24] Roncal C., Martínez-Aguilar E., Orbe J., Ravassa S., Fernandez-Montero A., Saenz-Pipaon G., Ugarte A., Mendoza A.E.-H.D., Rodriguez J.A., Fernández-Alonso S. (2019). Trimethylamine-N-Oxide (TMAO) Predicts Cardiovascular Mortality in Peripheral Artery Disease. Sci. Rep..

[25] Kanitsoraphan C., Rattanawong P., Charoensri S., Senthong V. (2018). Trimethylamine N-Oxide and Risk of Cardiovascular Disease and Mortality. Curr. Nutr. Rep..

[26] Wang Z., Klipfell E., Bennett B.J., Koeth R., Levison B.S., Dugar B., Feldstein A.E., Britt E.B., Fu X., Chung Y.-M. (2011). Gut Flora Metabolism of Phosphatidylcholine Promotes Cardiovascular Disease. Nature.

[27] Tang W.W., Wang Z., Levison B.S., Koeth R.A., Britt E.B., Fu X., Wu Y., Hazen S.L. (2013). Intestinal Microbial Metabolism of Phosphatidylcholine and Cardiovascular Risk. N. Engl. J..

[28] Senthong V., Wang Z., Li X.S., Fan Y., Wu Y., Tang W.H.W., Hazen S.L. (2016). Intestinal Microbiota-Generated Metabolite Trimethylamine-N-Oxide and 5-Year Mortality Risk in Stable Coronary Artery Disease: The Contributory Role of Intestinal Microbiota in a COURAGE-Like Patient Cohort. J. Am. Heart Assoc..

[29] Zeisel S.H., Warrier M. (2017). TrimethylamineN-Oxide, the Microbiome, and Heart and Kidney Disease. Annu. Rev. Nutr..

[30] Yoshida N., Yamashita T., Hirata K.-I. (2018). Gut Microbiome and Cardiovascular Diseases. Diseases.

[31] Trøseid M., Andersen G.Ø., Broch K., Hov J.R. (2020). The Gut Microbiome in Coronary Artery Disease and Heart Failure: Current Knowledge and Future Directions. EBioMedicine.

[32] Kazemian N., Mahmoudi M., Halperin F., Wu J.C., Pakpour S. (2020). Gut Microbiota and Cardiovascular Disease: Opportunities and Challenges. Microbiome.

[33] Tang W.W., Bäckhed F., Landmesser U., Hazen S.L. (2019). Intestinal Microbiota in Cardiovascular Health and Disease. J. Am. Coll. Cardiol..

[34] Sata Y., Marques F.Z., Kaye D.M. (2020). The Emerging Role of Gut Dysbiosis in Cardio-Metabolic Risk Factors for Heart Failure. Curr. Hypertens. Rep..

[35] Zhang D., Li S., Wang N., Tan H.-Y., Zhang Z., Feng Y. (2020). The Cross-Talk between Gut Microbiota and Lungs in Common Lung Diseases. Front. Microbiol..

[36] Loverdos K., Bellos G., Kokolatou L., Vasileiadis I., Giamarellos E., Pecchiari M., Koulouris N., Koutsoukou A., Rovina N. (2019). Lung Microbiome in Asthma: Current Perspectives. J. Clin. Med..

[37] Gao Q.Y., Chen Y.X., Fang J.Y. (2020). 2019 Novel Coronavirus Infection and Gastrointestinal Tract. J. Dig. Dis..

[38] Zuo T., Zhang F., Lui G.C., Yeoh Y.K., Li A.Y., Zhan H., Wan Y., Chung A., Cheung C.P., Chen N. (2020). Alterations in Gut Microbiota of Patients With COVID-19 During Time of Hospitalization. Gastroenterology.

[39] Lyon L. (2018). All disease begins in the gut’: Was Hippocrates right?. Brain.

[40] Carabotti M., Scirocco A., Maselli M.A., Severi C. (2015). The gut-brain axis: Interactions between enteric microbiota, central and enteric nervous systems. Ann. Gastroenterol..

[41] Mu C., Yang Y., Zhu W. (2016). Gut Microbiota: The Brain Peacekeeper. Front. Microbiol..

[42] Lach G., Schellekens H., Dinan T.G., Cryan J.F. (2017). Anxiety, Depression, and the Microbiome: A Role for Gut Peptides. Neurotherapeutics.

[43] Dinan T.G., Cryan J.F. (2013). Melancholic Microbes: A Link between Gut Microbiota and Depression?. Neurogastroenterol. Motil..

[44] Bermúdez-Humarán L.G., Salinas E., Ortiz G.G., Ramirez-Jirano L.J., Morales J.A., Bitzer-Quintero O.K. (2019). From Probiotics to Psychobiotics: Live Beneficial Bacteria Which Act on the Brain-Gut Axis. Nutrients.

[45] Cani P.D. (2018). Human Gut Microbiome: Hopes, Threats and Promises. Gut.

[46] Barko P., Mcmichael M., Swanson K., Williams D. (2017). The Gastrointestinal Microbiome: A Review. J. Vet. Intern. Med..

[47] Hills R.D., Pontefract B.A., Mishcon H.R., Black C.A., Sutton S.C., Theberge C.R. (2019). Gut Microbiome: Profound Implications for Diet and Disease. Nutrients.

[48] Zeevi D., Korem T., Zmora N., Israeli D., Rothschild D., Weinberger A., Ben-Yacov O., Lador D., Avnit-Sagi T., Lotan-Pompan M. (2015). Personalized Nutrition by Prediction of Glycemic Responses. Cell.

[49] Mendes-Soares H., Raveh-Sadka T., Azulay S., Edens K., Ben-Shlomo Y., Cohen Y., Ofek T., Bachrach D., Stevens J., Colibaseanu D. (2019). Assessment of a Personalized Approach to Predicting Postprandial Glycemic Responses to Food Among Individuals Without Diabetes. JAMA Netw. Open.

[50] Chen P.B., Black A.S., Sobel A.L., Zhao Y., Mukherjee P., Molparia B., Moore N.E., Muench G.R.A., Wu J., Chen W. (2020). Directed Remodeling of the Mouse Gut Microbiome Inhibits the Development of Atherosclerosis. Nat. Biotechnol..

[51] Sharma H. (2016). Ayurveda: Science of life, genetics, and epigenetics. AYU.

[52] Bordoni L., Gabbianelli R. (2019). Primers on nutrigenetics and nutri(epi)genomics: Origins and development of precision nutrition. Biochimie.

[53] Sharma H., Meade J.G. (2018). Dynamic DNA.

[54] Dash B., Sharma R.K. (1995). Charaka Samhita.

[55] Prasher B., Negi S., Aggarwal S., Mandal A.K., Sethi T.P., Deshmukh S.R., Purohit S.G., Sengupta S., Khanna S., Mohammad F. (2008). Whole Genome Expression and Biochemical Correlates of Extreme Constitutional Types Defined in Ayurveda. J. Transl. Med..

[56] Travis F.T., Wallace R.K. (2015). Dosha brain-types: A neural model of individual differences. J. Ayurveda Integr. Med..

[57] Chauhan N.S., Pandey R., Mondal A.K., Gupta S., Verma M.K., Jain S., Ahmed V., Patil R., Agarwal D., Girase B. (2018). Western Indian Rural Gut Microbial Diversity in Extreme Prakriti Endo-Phenotypes Reveals Signature Microbes. Front. Microbiol..

[58] Jnana A., Murali T.S., Guruprasad K.P., Satyamoorthy K. (2020). Prakriti phenotypes as a stratifier of gut microbiome: A new frontier in personalized medicine? [published online ahead of print, 2020 Jul 24]. J. Ayurveda Integr. Med..

[59] Peterson C.T., Denniston K., Chopra D. (2017). Therapeutic Uses of Triphala in Ayurvedic Medicine. J. Altern. Complement. Med..

[60] Aggarwal B.B., Yuan W., Li S., Gupta S.C. (2013). Curcumin-Free Turmeric Exhibits Anti-Inflammatory and Anticancer Activities: Identification of Novel Components of Turmeric. Mol. Nutr. Food Res..

[61] Mcfadden R.-M.T., Larmonier C.B., Shehab K.W., Midura-Kiela M., Ramalingam R., Harrison C.A., Besselsen D.G., Chase J.H., Caporaso J.G., Jobin C. (2015). The Role of Curcumin in Modulating Colonic Microbiota During Colitis and Colon Cancer Prevention. Inflamm. Bowel Dis..

[62] Nelson K.M., Dahlin J.L., Bisson J., Graham J., Pauli G.F., Walters M.A. (2017). The Essential Medicinal Chemistry of Curcumin. J. Med. Chem..

[63] Lopresti A.L. (2018). The Problem of Curcumin and Its Bioavailability: Could Its Gastrointestinal Influence Contribute to Its Overall Health-Enhancing Effects?. Adv. Nutr..

[64] Shen L., Liu L., Ji H.-F. (2017). Regulative Effects of Curcumin Spice Administration on Gut Microbiota and Its Pharmacological Implications. Food Nutr. Res..

[65] Di Meo F., Margarucci S., Galderisi U., Crispi S., Peluso G. (2019). Curcumin, Gut Microbiota, and Neuroprotection. Nutrients.

[66] Guruprasad K.P., Dash S., Shivakumar M.B., Shetty P.R., Raghu K.S., Shamprasad B.R., Udupi V., Acharya R.V., Vidya P.B., Nayak J. (2017). Influence of Amalaki Rasayana on Telomerase Activity and Telomere Length in Human Blood Mononuclear Cells. J. Ayurveda Integr. Med. Med..

[67] Agrawal A.K., Yadav C.R., Meena M.S. (2010). Physiological aspects of Agni. AYU.

[68] Sharma H. (2009). Leaky gut syndrome, dysbiosis, ama, free radicals, and natural antioxidants. AYU.

[69] Fasano A. (2012). Zonulin, Regulation of Tight Junctions, and Autoimmune Diseases. Ann. N. Y. Acad. Sci..

[70] Sturgeon C., Fasano A. (2016). Zonulin, a Regulator of Epithelial and Endothelial Barrier Functions, and Its Involvement in Chronic Inflammatory Diseases. Tissue Barriers.

[71] Kelly J.R., Kennedy P.J., Cryan J.F., Dinan T.G., Clarke G., Hyland N.P. (2015). Breaking down the barriers: The gut microbiome, intestinal permeability and stress-related psychiatric disorders. Front. Cell. Neurosci..

[72] Fasano A. (2020). All disease begins in the (leaky) gut: Role of zonulin-mediated gut permeability in the pathogenesis of some chronic inflammatory diseases. F1000Research.

[73] Kecklund G., Axelsson J. (2016). Health Consequences of Shift Work and Insufficient Sleep. BMJ.

[74] Zarrinpar A., Chaix A., Panda S. (2016). Daily Eating Patterns and Their Impact on Health and Disease. Trends Endocrinol. Metab..

[75] Thaiss C.A., Zeevi D., Levy M., Zilberman-Schapira G., Suez J., Tengeler A.C., Abramson L., Katz M.N., Korem T., Zmora N. (2014). Transkingdom Control of Microbiota Diurnal Oscillations Promotes Metabolic Homeostasis. Cell.

[76] Kaczmarek J.L., Thompson S.V., Holscher H.D. (2017). Complex interactions of circadian rhythms, eating behaviors, and the gastrointestinal microbiota and their potential impact on health. Nutr. Rev..

[77] Smits S.A., Leach J., Sonnenburg E.D., Gonzalez C.G., Lichtman J.S., Reid G., Knight R., Manjurano A., Changalucha J., Elias J.E. (2017). Seasonal Cycling in the Gut Microbiome of the Hadza Hunter-Gatherers of Tanzania. Science.

[78] Davenport E.R., Mizrahi-Man O., Michelini K., Barreiro L.B., Ober C., Gilad Y. (2014). Seasonal Variation in Human Gut Microbiome Composition. PLoS ONE.

[79] Deaver J.A., Eum S.Y., Toborek M. (2018). Circadian Disruption Changes Gut Microbiome Taxa and Functional Gene Composition. Front. Microbiol..

[80] Voigt R., Forsyth C., Green S., Engen P., Keshavarzian A. (2016). Circadian Rhythm and the Gut Microbiome. Int. Rev. Neurobiol..

[81] Paulose J.K., Wright J.M., Patel A.G., Cassone V.M. (2016). Human Gut Bacteria Are Sensitive to Melatonin and Express Endogenous Circadian Rhythmicity. PLoS ONE.

[82] Reynolds A.C., Paterson J.L., Ferguson S.A., Stanley D., Wright K.P., Dawson D. (2017). The Shift Work and Health Research Agenda: Considering Changes in Gut Microbiota as a Pathway Linking Shift Work, Sleep Loss and Circadian Misalignment, and Metabolic Disease. Sleep Med. Rev..

[83] Wallace R.K., Wallace S., Stenberg S., Davis J., Farley A. (2019). The Rest and Repair Diet.

[84] Perlmutter D., Loberg K. (2015). The Brain Maker: The Power of Gut Microbes to Heal and Protect Your Brain for Life.

[85] Kadus P.A., Vedpathak S.M. (2017). Comparative Study of Anuvasana Basti with Constant and Escalating Dose as an Alternative to Snehapana in Purvakarma of Vamana and Virechana. J. Ayurveda Integr. Med..

[86] Anu M., Kunjibettu S., Archana S., Dei L. (2017). Management of Premature Contractions with Shatavaryadi Ksheerapaka Basti—A Case Report. AYU.

[87] Pooja B., Bhatted S. (2016). A Standard Controlled Clinical Study on Virechana Karma and Lekhana Basti in the Management of Dyslipidemia (Medoroga). AYU.

[88] Kadus P., Vedpathak S. (2014). Anuvasan Basti in Escalating Dose Is an Alternative for Snehapana before Vamana and Virechana: Trends from a Pilot Study. J. Ayurveda Integr. Med..

[89] Shukla G., Bhatted S., Dave A., Shukla V. (2013). Efficacy of Virechana and Basti Karma with Shamana Therapy in the Management of Essential Hypertension: A Comparative Study. AYU.

[90] Auti S., Thakar A., Shukla V., Ravishankar B. (2013). Assessment of Lekhana Basti in the Management of Hyperlipidemia. AYU.

[91] Swapnil S., Anup B., Ashok B., Ravishankar B., Shukla V. (2013). Evaluation of Anti-Hyperlipidemic Activity of Lekhana Basti in Albino Rats. AYU.

[92] Baria R., Pandya D., Joshi N. (2011). Clinical Efficacy of Panchamuladi Kaala Basti (Enema) in the Management of Amavata (Rheumatoid Arthritis). AYU.

[93] Periasamy S., Hsu D.-Z., Chandrasekaran V.R.M., Liu M.-Y. (2012). Sesame Oil Accelerates Healing of 2,4,6-Trinitrobenzenesulfonic Acid–Induced Acute Colitis by Attenuating Inflammation and Fibrosis. J. Parenter. Enteral. Nutr..

[94] Hou R., Lin M., Wang M., Tzen J. (2003). Increase of Viability of Entrapped Cells of Lactobacillus Delbrueckii Ssp. Bulgaricus in Artificial Sesame Oil Emulsions. J. Dairy Sci..

[95] Peterson C.T., Lucas J., John-Williams L.S., Thompson J.W., Moseley M.A., Patel S., Peterson S.N., Porter V., Schadt E.E., Mills P.J. (2016). Identification of Altered Metabolomic Profiles Following a Panchakarma-Based Ayurvedic Intervention in Healthy Subjects: The Self-Directed Biological Transformation Initiative (SBTI). Sci. Rep..

[96] Cani P.D., Knauf C. (2016). How Gut Microbes Talk to Organs: The Role of Endocrine and Nervous Routes. Mol. Metab..

[97] Herr N., Bode C., Duerschmied D. (2017). The Effects of Serotonin in Immune Cells. Front. Cardiovasc. Med..

[98] Boyer E.W., Shannon M. (2005). The serotonin syndrome. N. Engl. J. Med..

[99] Frick A., Åhs F., Engman J., Jonasson M., Alaie I., Björkstrand J., Frans Ö., Faria V., Linnman C., Appel L. (2015). Serotonin Synthesis and Reuptake in Social Anxiety Disorder. JAMA Psychiatry.

[100] Corrêa-Oliveira R., Fachi J.L., Vieira A., Sato F.T., Vinolo M.A.R. (2016). Regulation of Immune Cell Function by Short-Chain Fatty Acids. Clin. Transl. Immunol..

[101] Foster J.A., Rinaman L., Cryan J.F. (2017). Stress & the Gut-Brain Axis: Regulation by the Microbiome. Neurobiol. Stress.

[102] Wallace R.K. (1970). Physiological Effects of Transcendental Meditation. Science.

[103] Wallace R., Benson H., Wilson A. (1971). A Wakeful Hypometabolic Physiologic State. Am. J. Physiol..

[104] Travis F., Shear J. (2010). Focused Attention, Open Monitoring and Automatic Self-Transcending: Categories to Organize Meditations from Vedic, Buddhist and Chinese Traditions. Conscious. Cogn..

[105] Nidich S., Mills P.J., Rainforth M., Heppner P., Schneider R.H., Rosenthal N.E., Salerno J., Gaylord-King C., Rutledge T. (2018). Non-Trauma-Focused Meditation versus Exposure Therapy in Veterans with Post-Traumatic Stress Disorder: A Randomised Controlled Trial. Lancet Psychiatry.

[106] Barnes V., Orme-Johnson D. (2006). Clinical and Pre-Clinical Applications of the Transcendental Meditation Program^®^ in the Prevention and Treatment of Essential Hypertension and Cardiovascular Disease in Youth and Adults. Curr. Hypertens Rev..

[107] Schneider R.H., Grim C.E., Rainforth M.V., Kotchen T., Nidich S.I., Gaylord-King C., Salerno J.W., Kotchen J.M., Alexander C.N. (2012). Stress Reduction in the Secondary Prevention of Cardiovascular Disease. Circ. Cardiovasc. Qual. Outcomes.

[108] Tillisch K., Mayer E.A., Gupta A., Gill Z., Brazeilles R., Nevé B.L., Vlieg J.E.V.H., Guyonnet D., Derrien M., Labus J.S. (2017). Brain Structure and Response to Emotional Stimuli as Related to Gut Microbial Profiles in Healthy Women. Psychosom. Med..

[109] Tillisch K., Labus J., Kilpatrick L., Jiang Z., Stains J., Ebrat B., Guyonnet D., Legrain–Raspaud S., Trotin B., Naliboff B. (2013). Consumption of Fermented Milk Product With Probiotic Modulates Brain Activity. Gastroenterology.

[110] Wallace R.K., Wallace S. (2017). Gut Crisis.

